# Noninvasive continuous blood pressure prediction using FlexNIRS and machine learning during carotid endarterectomy

**DOI:** 10.1117/1.JBO.30.S2.S23913

**Published:** 2025-09-19

**Authors:** Zahra Einalou, Mehrdad Dadgostar, Kuan-Cheng Wu, Alyssa Martin, Mitchell B. Robinson, Marco Renna, Jason Z. Qu, John Sunwoo, Maria Angela Franceschini

**Affiliations:** a Massachusetts General Hospital, Harvard Medical School, Athinoula A. Martinos Center for Biomedical Imaging, Boston, Massachusetts, United States; b Massachusetts General Hospital, Harvard Medical School, Department of Anesthesia, Critical Care and Pain Medicine, Boston, Massachusetts, United States

**Keywords:** noninvasive blood pressure monitoring, near-infrared spectroscopy photoplethysmography, FlexNIRS, linear regression model, Gaussian process regression, carotid endarterectomy

## Abstract

**Significance:**

Continuous blood pressure (BP) monitoring is crucial for maintaining hemodynamic stability and complication prevention. Near-infrared spectroscopy photoplethysmography (NIRS-PPG) offers a noninvasive alternative to arterial lines (A-line) for continuous BP monitoring.

**Aim:**

We aim to assess whether a wearable NIRS-PPG device (FlexNIRS) can estimate mean arterial pressure (MAP) using linear and Gaussian process regression (GPR) models.

**Approach:**

NIRS-PPG signals were recorded bilaterally in 10 patients undergoing carotid endarterectomy. Subject-specific linear regression and GPR models predicted MAP based on heart rate and peak features of the NIRS-PPG signal. A-line readings served as the reference.

**Results:**

All models achieved strong performance with $R^{2}\geq 0.75$. The two-feature GPR model improved accuracy ($R^{2}=0.78$), whereas adding a third feature further enhanced performance ($R^{2}=0.82$). Improvements in $R^{2}$, mean absolute error, and root mean squared error were statistically significant. The highest accuracy was observed contralateral to the surgical site using the 2.8-cm source-detector separation.

**Conclusions:**

This preliminary study supports the feasibility of noninvasive MAP estimation using NIRS-PPG and machine learning. The approach may provide a practical alternative for BP monitoring after A-line removal in postoperative and intensive care unit settings.

## 1 Introduction

Carotid endarterectomy (CEA) is a surgical procedure performed to remove atherosclerotic plaque from the carotid artery to reduce stroke risk and restore adequate blood flow to the brain.^1^^,^^2^ During the operation, the carotid artery is temporarily clamped to stop blood flow, whereas the plaque is removed and then unclamped to restore circulation. Continuous blood pressure (BP) monitoring is essential to ensure adequate cerebral perfusion during artery clamping and to prevent hypertension-related complications after unclamping.^3^ BP modulation under clinical supervision throughout the procedure helps minimize neurological and cardiovascular risks.^4^ Intraoperatively, BP is typically measured using an arterial line (A-line), which offers continuous and accurate measurements.^5^ However, the A-line is removed post-surgery, and patients are usually discharged the next day. This creates a critical gap in monitoring during the early postoperative period, when the risk of cerebral hyperperfusion syndrome—marked by excessive cerebral blood flow, stroke, and other severe complications—is highest.^6^ A noninvasive, continuous BP monitoring solution during recovery is therefore crucial to enhance patient safety and outcomes.

This issue is not unique to CEA but applies more broadly to transitions from critical care to the observation phase, when the A-line is removed due to its invasive nature and associated risks, such as infection or thrombosis. At that point, only intermittent BP monitoring remains. This reduction in monitoring resolution can delay the detection of hemodynamic instability during a period of elevated risk.

Several noninvasive continuous BP monitoring methods, including volume-clamp devices such as Finapres,^7^^,^^8^ photoplethysmography (PPG) pulse transit time,^9^ tonometry,^10^ and pulse wave velocity (PWV),^11^ offer unique advantages and limitations. Finapres provides continuous BP readings but can become uncomfortable over time and is prone to drift, requiring frequent recalibration. PPG, commonly measured at the wrist or finger, is widely available and cost-effective, but is sensitive to motion artifacts and peripheral perfusion variability.^12^ Tonometry and PWV allow continuous measurements with reasonable accuracy but depend on precise sensor placement and are affected by local arterial stiffness and motion artifacts.

To overcome these limitations, we introduce near-infrared spectroscopy-photoplethysmography (NIRS-PPG), implemented using the FlexNIRS device,^13^ for large-separation reflection PPG measurements on the forehead.

We use the term NIRS-PPG to distinguish our method from standard PPG, which is typically acquired at peripheral sites such as the finger. Our signal is recorded using a continuous-wave near-infrared spectroscopy (CW-NIRS) device, originally developed for cerebral monitoring but adapted here to capture pulsatile waveforms from the forehead using a single wavelength (850 nm). We selected 850 nm because it is more sensitive to oxyhemoglobin (HbO) than the alternative wavelength available (760 nm), and it provides stronger pulsatile signals, reflecting the fact that arterial blood is nearly fully oxygenated (98% to 100%). In addition, the use of a reflection geometry with 2 to 3 cm source-detector separation requires a NIRS device, as conventional pulse oximeters lack sufficient signal-to-noise ratio at this depth. Unlike standard NIRS applications, we do not estimate hemoglobin concentrations or analyze the slow component of the signal (0.01 to 0.5 Hz). Instead, we focus on the pulsatile waveform (∼1  Hz), extracting features to estimate blood pressure. This approach leverages the hardware and depth advantages of NIRS while clearly differing from both standard PPG and oxygenation-focused NIRS techniques.

FlexNIRS is a flexible, wearable NIRS device that, in addition to measuring cerebral oximetry, provides high-resolution PPG signals (266 Hz) at large source-detector separations (2 to 3 cm).^13^ This enables robust acquisition from the forehead, a site less affected by peripheral vasoconstriction, low perfusion, and motion artifacts.^13^^,^^14^ When calibrated using reference BP measurements and combined with machine learning methods, FlexNIRS has the potential to enable continuous, noninvasive blood pressure monitoring across a variety of clinical settings.

Recent studies have shown that machine learning models can effectively predict BP changes during the postoperative period, enhancing patient care by enabling timely interventions and preventing potential complications.^15^^,^^16^

In this study, we compare a simple linear regression model with Gaussian process regression (GPR)—a nonlinear machine learning approach—to predict continuous, noninvasive BP during CEA. The linear model provides a straightforward and interpretable estimation of mean arterial pressure (MAP) based on selected features. This choice allows us to directly assess the physiological relevance of individual features and to establish a clear baseline for comparison. In contrast, GPR captures more complex relationships in the data and incorporates uncertainty estimates, potentially improving predictive accuracy.^17^ This dual-model approach highlights the trade-off between the interpretability and simplicity of linear regression and the flexibility and predictive performance of GPR.

In addition, we aim to overcome the limitations of traditional finger or wrist PPG methods by providing continuous monitoring from the forehead, a site that is less affected by peripheral vasoconstriction, low perfusion, and motion artifacts. Unlike the finger, the forehead experiences more stable blood flow, as it is less influenced by peripheral vascular tone—particularly in conditions such as cold exposure or hypotension.^14^ This stability is due in part to supply from the carotid artery, which is more tightly autoregulated and less susceptible to local perfusion variability. NIRS-PPG measurements from the forehead also better reflect central blood pressure, given the proximity of vessels such as the supraorbital and frontal arteries to central circulation. A recent study has confirmed that central pressures can be inferred more accurately from the head and neck vasculature than from peripheral sites, due to reduced pressure amplification and vascular compliance differences.^18^ Together, these factors make the forehead a robust and clinically advantageous site for NIRS-PPG-based blood pressure monitoring.

Validation using the data acquired during CEA procedures is effective due to the significant BP variations during the procedure, as the patient’s blood pressure is deliberately modulated, with increases during the clamping phase to promote collateral blood flow and decreases during unclamping to prevent excessive cerebral blood flow and protect the sutured vessels. In addition, the vascular resistance on the ipsilateral side—the same side as the carotid artery undergoing clamping—changes dramatically due to clamping (temporary occlusion) and unclamping of the carotid artery, significantly altering the cerebral perfusion. These combined BP and hemodynamic challenges create a demanding environment for testing the accuracy and adaptability of FlexNIRS for continuous BP monitoring. It is important to note that this study represents a preliminary feasibility assessment, intended to evaluate the potential of NIRS-PPG under ideal ground-truth conditions before broader validation in larger and more diverse populations.

## 2 Materials and Methods

### 2.1 Study Patients and Procedure

This study was approved by the Institutional Review Board of Mass General Brigham (MGB), protocol 2022P003013, and conducted at Massachusetts General Hospital (MGH) between May and December 2023. Eligible patients scheduled for CEA surgery were identified and contacted by the study team. Patients interested in participating were informed about the study, and those meeting the inclusion criteria provided written informed consent. A total of eleven patients consented to participate and were monitored during surgery. Ten of these patients (mean age 69.6±8.3 years, range 57 to 80, 2 females) were included in this study. One patient had a pacemaker resulting in a constant heart rate. One patient was excluded due to an issue with the continuous A-line BP recording. Table 1 provides details on the demographics, surgical side, and measurement duration of the 10 subjects.

**Table 1:** Patient population and measurement characteristics.

Patient	Age	Sex	Surgery side	Missing data	Measurement duration
1	73	M	R	—	3 h, 23 min
2	72	F	R	L, contralateral	2 h, 27 min
3	57	M	R	—	3 h, 5 min
4	80	M	R	—	4 h, 40 min
5	68	F	L	—	2 h, 34 min
6	58	M	R	—	5 h, 42 min
7	76	M	R	—	4 h, 21 min
8	70	M	R	R, ipsilateral	2 h, 5 min
9	80	M	L	—	4 h, 5 min
10	62	M	R	R, ipsilateral	3 h, 44 min
Age, years (mean ± SD)			69.60 ± 8.35		
Range			57 to 80		

F, female; M, male; SD, standard deviation; L, left; R, right.

**Fig. 1:**
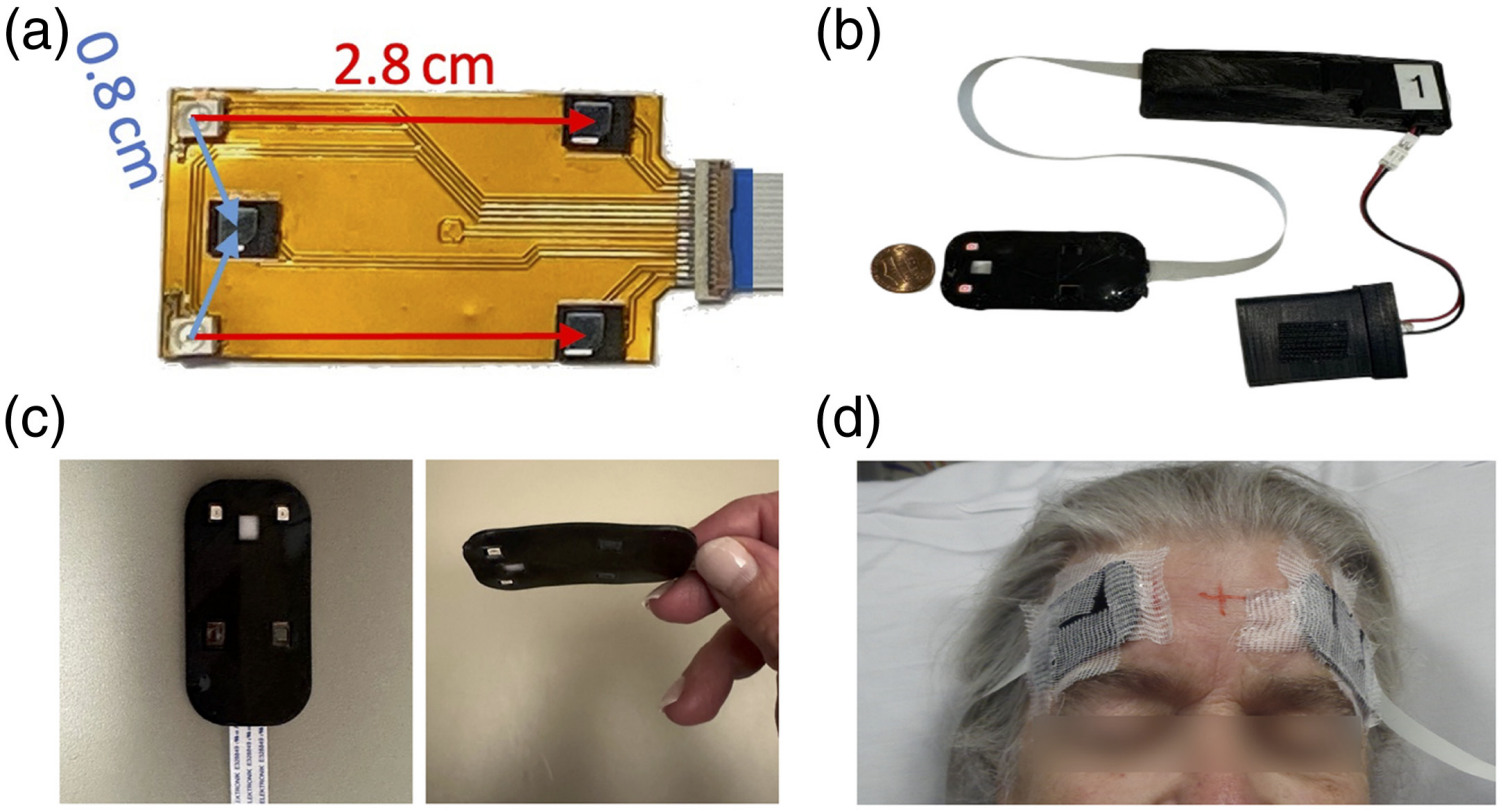
(a) FlexNIRS sensor without cover, showing the geometry of the light sources (white squares) and detectors (black rectangles); (b) one of the FlexNIRS device used in this study, including the sensor, control unit, and battery; (c) close-up of the FlexNIRS sensor embedded in a flexible 3D-printed sleeve; and (d) sensors attached to the patient’s forehead during preoperative procedures.

The study utilized the Generation 2 FlexNIRS system, a continuous-wave, open-source, low-cost, and wearable wireless cerebral oximeter.^13^ Compared with the original device, Gen 2 uses positive-intrinsic-negative (PIN) photodiodes with an optical long pass filter with a cut-on at 700 nm to reduce interference from the strong ambient light of the surgical lamps. As a result, the 735 nm LEDs were replaced by 760 nm LEDs to reduce attenuation. In addition, Gen 2 allows for a 266-Hz sampling rate, acquiring data at 850 and 760 nm, with three detectors symmetrically arranged to achieve two sets of source-detector separations at 0.8, 2.8, and 3.3 cm. To focus on the arterial component of the pulsatile signal for blood pressure analysis, we used data only from the 850-nm wavelength, which provides larger NIRS-PPG signals due to the higher absorption by oxygenated hemoglobin compared with 760 nm. We analyzed data from the 0.8- and 2.8-cm source-detector separations, which offered higher signal-to-noise ratios (SNR). Because our goal was to extract pulsatile features rather than quantify hemoglobin concentrations, computing oxy- and deoxyhemoglobin using multiple wavelengths would have introduced unnecessary noise and was not required for this application. Two FlexNIRS sensors were attached to opposite sides of the patient’s forehead, as far apart as possible to avoid optical crosstalk, alongside the clinically used electroencephalogram (EEG) electrodes during preoperative procedures (Fig. 1). Collodion was used to secure the FlexNIRS sensors on the forehead. In three subjects, we obtained only unilateral FlexNIRS recordings due to technical issues (see Table 1).

Measurements were conducted in an operating room under general anesthesia, starting from the induction of anesthesia and continuing until its cessation. During this period, we continuously acquired the NIRS-PPG (via FlexNIRS), continuous BP (via an A-line), electrocardiogram (ECG), and EEG. Although EEG electrodes were placed as part of the clinical monitoring protocol, EEG data were not analyzed in this study.

### 2.2 Pre-processing of Data

**Fig. 2:**
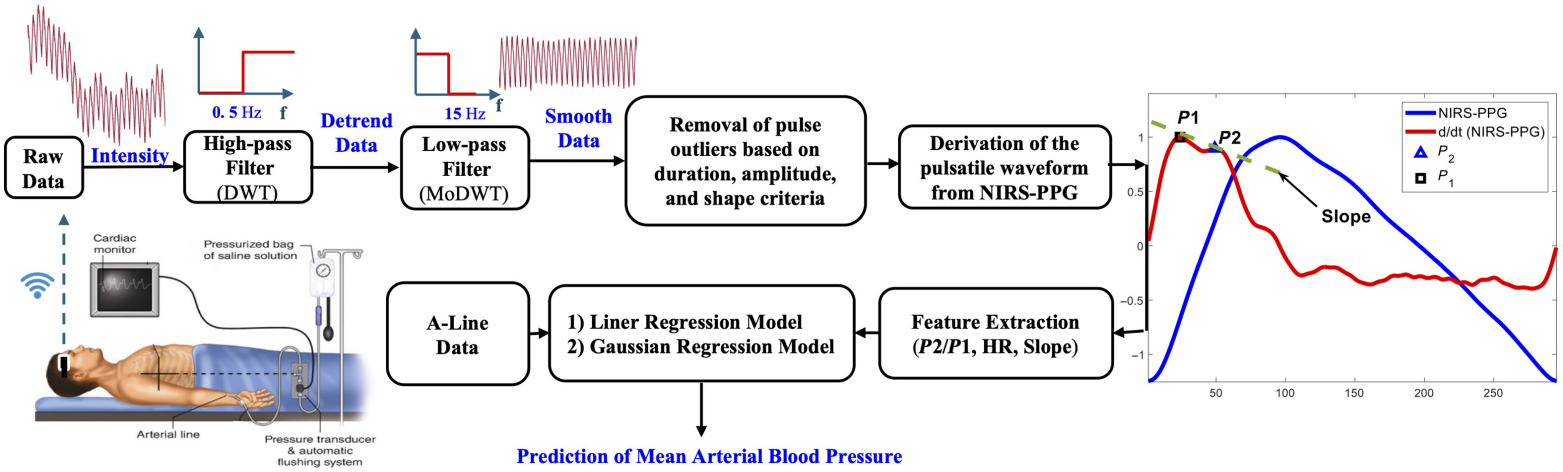
Schematic representation of the continuous blood pressure estimation methodology, including data collection, signal preprocessing, feature extraction, and machine learning. The process begins with data collection from CEA patients. Preprocessing steps are then applied to remove motion artifacts and noisy segments, including the use of a band-pass filter. Next, features such as the P2/P1 ratio, heart rate (HR), and the slope between P1 and P2 are extracted. Finally, the data is split into training, validation, and testing sets for the model development. *DWT, discrete wavelet transform; MODWT, maximal overlap discrete wavelet transform; P1, first systolic peak; P2, second peak.

Figure 2 depicts the complete architecture of our continuous blood pressure estimation method as a flow chart. All signal processing, feature extraction, modeling, and statistical analyses were performed using MATLAB version 2024a.

To extract pulsatile waveforms from the FlexNIRS data, we subtracted ambient light from the raw intensity signal acquired at 266 Hz. Ambient light was measured once per cycle, every 3.76 ms during periods when no LEDs were active and digitally subtracted in post-processing, as described in the FlexNIRS paper.^13^ Data segments affected by motion artifacts, detected by the accelerometer (ACC), or by excessive ambient light leakage, typically occurring before surgical draping, were removed. Subject-specific percentiles accounted for individual variations in motion and ambient light exposure.

We applied band-pass filtering using a combination of three-level discrete wavelet transform (DWT) and the maximal overlap discrete wavelet transform (MODWT), both with Daubechies (db5) mother wavelets at eight decomposition levels. The choice of mother wavelet is crucial, as a closer match to the original signal improves both decomposition and reconstruction, enhancing feature extraction reliability.^19^ The DWT served as a high-pass filter with a cutoff frequency of 0.5 Hz to remove low-frequency systemic signals such as respiration and Mayer waves. MODWT was then applied as a low-pass filter with a cutoff of 15 Hz to eliminate high-frequency noise. We selected the 15 Hz cutoff because it captures the full bandwidth of arterial pulsations, including key features such as the systolic upstroke and dicrotic notch. Higher cutoffs (e.g., up to 25 Hz) were tested but did not improve feature stability or prediction accuracy. This wavelet-based band-pass filtering approach effectively isolated the NIRS-PPG components within the 0.5 to 15 Hz range, optimizing signal fidelity for further analysis.

Following filtering, we calculated the delta optical density (ΔOD) at 850 nm, using the formula $\Delta \mathrm{OD}=\ln[I_{0}/I(t)]$, where $I_{0}$ is the baseline light intensity. The resulting signals were then normalized using the interquartile range (25th to 75th percentiles).

NIRS-PPG outliers were identified and removed based on pulse duration, amplitude, and shape. For each pulse, duration and amplitude were calculated after removing slow baseline fluctuations using a 15-s moving median filter, which helped isolate transient features of the NIRS-PPG waveform. Outliers were defined using fixed percentile thresholds applied to all datasets: the 1st and 99th percentiles for pulse duration, and the 5th and 95th percentiles for amplitude, excluding pulses with unusually short/long durations or very low/high amplitudes. To detect shape outliers, we used the 7th and 93rd percentiles, based on shape similarity over 20 consecutive pulses. Pulses with suboptimal shapes were rejected to preserve physiologically relevant signals and minimize noise and artifacts. To further improve signal quality, we computed the average of four pulses with a 50% overlap, enhancing robustness against residual noise.

### 2.3 Feature Extraction

**Fig. 3:**
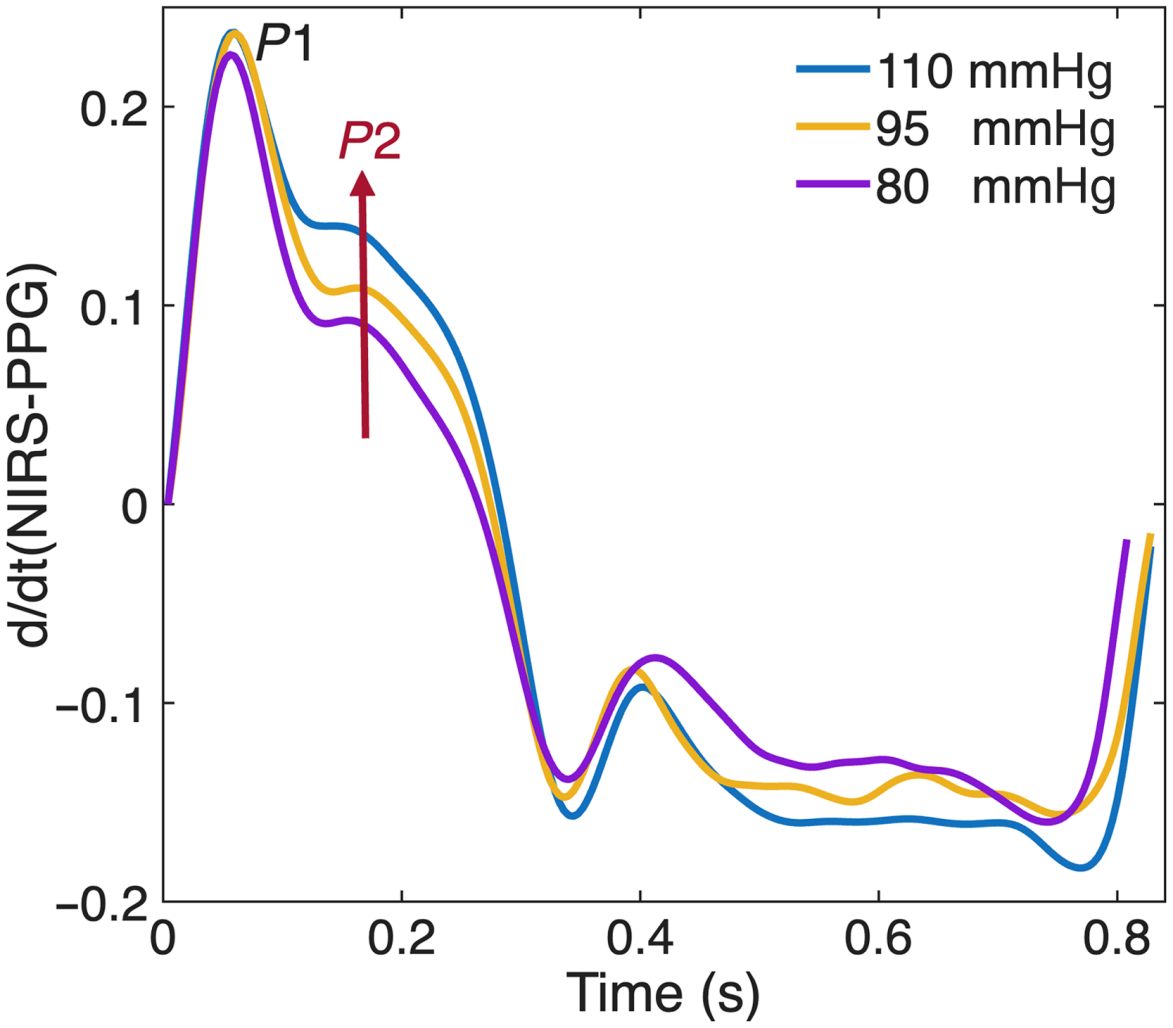
Normalized NIRS-PPG waveforms from patient #1, aligned to the first systolic peak (P1), illustrate changes in the relative amplitude of the second peak (P2) across varying blood pressure levels. As blood pressure decreases, P2 becomes progressively less pronounced and more difficult to identify.

The NIRS-PPG ΔOD signal is directly proportional to pulsatile changes in arterial blood volume, as light absorption increases with the expansion of blood-filled vessels during systole.^20^^,^^21^ Its first time derivative, d(NIRS−PPG)/dt, reflects the rate of change in blood volume and correlates with pulsatile blood flow.^19^^,^^21^^–^^23^ From this derivative, we extracted features sensitive to BP changes. Specifically, we calculate the ratio of the second peak (P2) to the first systolic peak (P1), which reflects the interaction between the forward (P1) and reflected (P2) pressure waves. A higher P2/P1 ratio indicates greater wave reflection, often linked to elevated blood pressure and enhanced sympathetic activity.^20^ This makes the P2/P1 ratio a physiologically meaningful, noninvasive marker of BP variation (Fig. 3).

In addition, we extracted heart rate (HR) to quantify cardiac cycle frequency and calculated the slope between P1 and P2 to capture the rate of transition between the two pressure waves, providing further insight into hemodynamic dynamics. Importantly, these features—P2/P1 ratio, HR, and P1 to P2 slope—are independent of pulse amplitude, allowing their use on both the ipsilateral and contralateral sides, even when pulse ipsilateral amplitude is strongly attenuated by surgical clamping.

To reduce the impact of noise and motion artifacts, we computed features over sliding windows that averaged four cardiac cycles, yielding a typical temporal resolution of ∼4  s, depending on heart rate. This window length was selected based on empirical testing, which showed that averaging across four beats produced the most stable and robust predictions. As for feature selection, in this work, we focused on the region near the systolic upstroke—particularly around the P1 and P2 peaks—as these features are most sensitive to arterial pressure and less influenced by venous return, vascular compliance, or local vascular variability.

The systolic peak is relatively easy to identify, whereas P2 can be less distinct and more challenging to detect. To improve P2 identification, we fit a combination of four Gaussian functions to the first time derivative of the NIRS-PPG signal. Each Gaussian is defined by its amplitude (A), mean (μ), and standard deviation (σ)—with parameters optimized to best match the observed peaks. The full model is shown in Eq. (1).

$$f(x)=G_{1}(x)+G_{2}(x)+G_{3}(x)+G_{4}(x),$$ (1)

where $G_{i}(x)=A_{i}e^{\frac{-(x-\mu_{i}{)}^{2}}{2\sigma_{i}^{2}}}$.

**Fig. 4:**
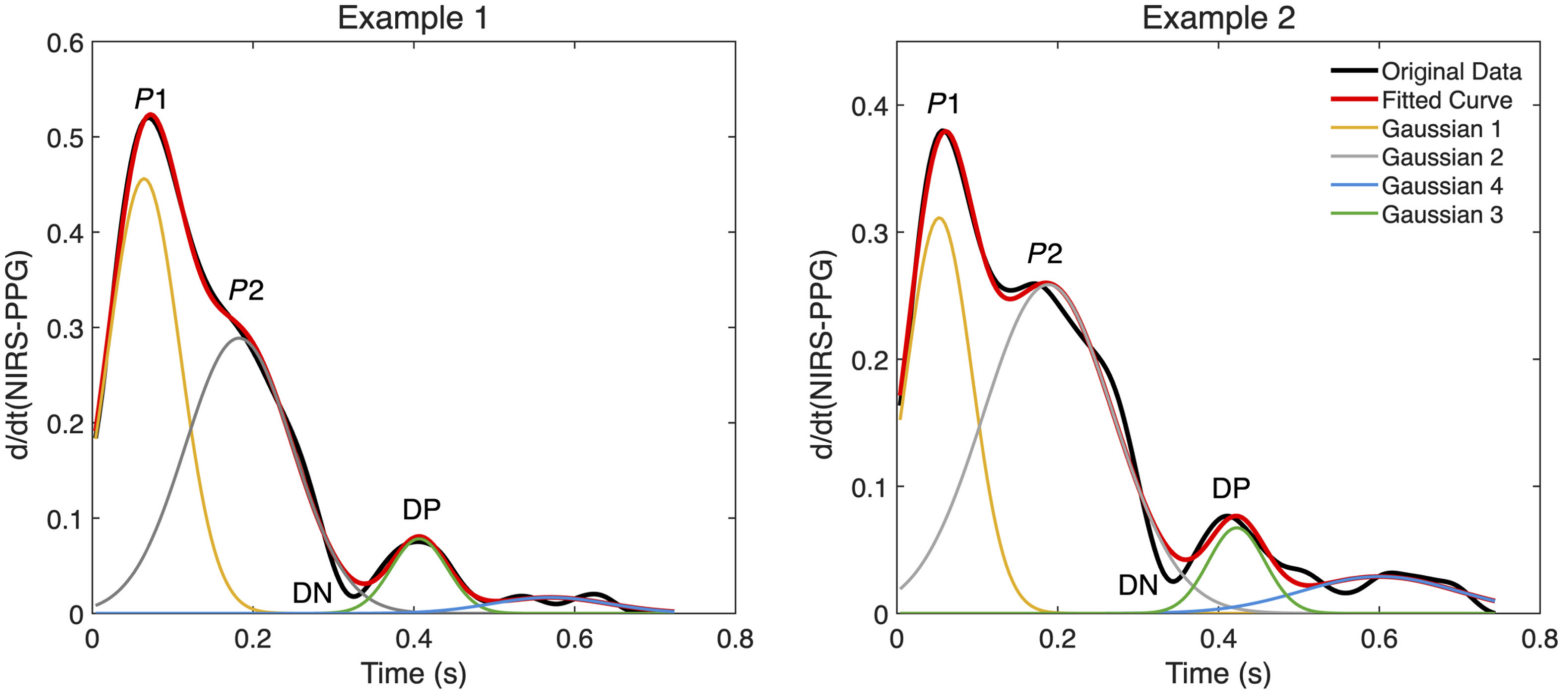
Identification of P2 using a four-Gaussian fit model. Four Gaussian functions are fitted to the first time derivative of the NIRS-PPG signal to identify key peaks: the primary systolic peak (P1), the reflected peak (P2), the diastolic peak (DP), and the fourth peak.

First, the initial Gaussian function is fitted to the first derivative of the NIRS-PPG signal to identify the primary systolic peak, P1. A second Gaussian is then applied to capture secondary or less prominent peaks that may be obscured in the raw signal. The dicrotic notch (DN), a small dip following the second peak, is identified next, and a third Gaussian is fitted in this region to locate the dicrotic peak (DP). Finally, a fourth Gaussian captures the last significant peak in the waveform, typically occurring after the DP (see Fig. 4).

A nonlinear optimization algorithm is used to fit the Gaussian functions to the first-derivative signal, adjusting their amplitude, mean, and standard deviation to minimize the difference between the fitted model and the actual data. After fitting, the peak positions (means) are extracted and sorted by time. P2 is identified as the second peak in the time-ordered sequence of fitted Gaussian means.

### 2.4 Regression Models

We employed two regression models to predict MAP. The same models were also applied separately to predict systolic BP (SBP) and diastolic BP (DBP). Predictive performance was evaluated using the coefficient of determination ($R^{2}$), derived from the linear fit between the predicted and actual values, and Bland-Altman limits of agreement.

To establish a baseline and provide interpretability, we used a simple linear regression model, given the approximately linear relationship observed between P2/P1 and BP in our dataset. In addition, we implemented GPR to capture nonlinear relationships and better model subject-specific variability.^24^^,^^25^

#### 2.4.1 Linear regression model

This model was constructed by fitting the P2/P1 ratio and HR to the target blood pressure metrics (MAP or SBP, DBP). For MAP prediction, the model is expressed as

$$\mathrm{MAP}=A\cdot \frac{P2}{P1}+B\cdot \mathrm{HR}+\epsilon ,$$ (2)

where A and B are the regression coefficients for the P2/P1 ratio and HR, respectively, and ϵ represents the DC blood pressure offset. These coefficients were obtained for each subject.

The standard error (SE) of each regression coefficient was calculated based on the residual variance of the model and the structure of the design matrix. For each subject, a linear regression model was fit using P2/P1 and HR as predictors of MAP. The residual variance was computed as the mean squared error between the observed and predicted MAP values, adjusted for the degrees of freedom. The SE for each coefficient (intercept, P2/P1, and HR) was then derived from this residual variance and the corresponding diagonal elements of the inverse of the matrix product $X^{T}X$, where X is the design matrix of predictors. This approach quantifies the uncertainty in each coefficient estimate and reflects how reliably each predictor contributes to MAP estimation for individual subjects.

#### 2.4.2 Gaussian process regression model

The GPR model is a nonparametric Bayesian approach commonly used for regression tasks. It provides a probabilistic framework capable of capturing complex, nonlinear relationships while quantifying predictive uncertainty.^17^ In this study, a separate GPR model was trained to predict each blood pressure metric (MAP, SBP, or DBP) one at a time.

For each subject, we selected a 20-min data segment with pronounced BP fluctuations—typically during unclamping and occasionally during clamping—for training and tuning. Within this segment, we applied five-fold cross-validation to optimize model parameters. For the GPR model, this included kernel hyperparameters (e.g., length scale and signal variance), tuned via marginal likelihood maximization. The remaining data were held out for out-of-sample testing, ensuring a clear temporal separation to minimize overfitting and data leakage.

The GPR model used a radial basis function (RBF) kernel to capture smooth, nonlinear dependencies. We first trained a two-feature model using the P2/P1 ratio and HR, consistent with the linear regression baseline. We then trained a three-feature model that included the P2–P1 slope to assess its additional predictive value.

Following training, the optimized GPR model was applied to the test data to predict each BP metric using the posterior mean, whereas the posterior variance quantified predictive uncertainty.

Performance was assessed using the coefficient of determination ($R^{2}$), root mean squared error (RMSE), mean absolute error (MAE), and Bland–Altman limits of agreement.

## 3 Results

### 3.1 MAP Prediction: Linear Regression Model

**Fig. 5:**
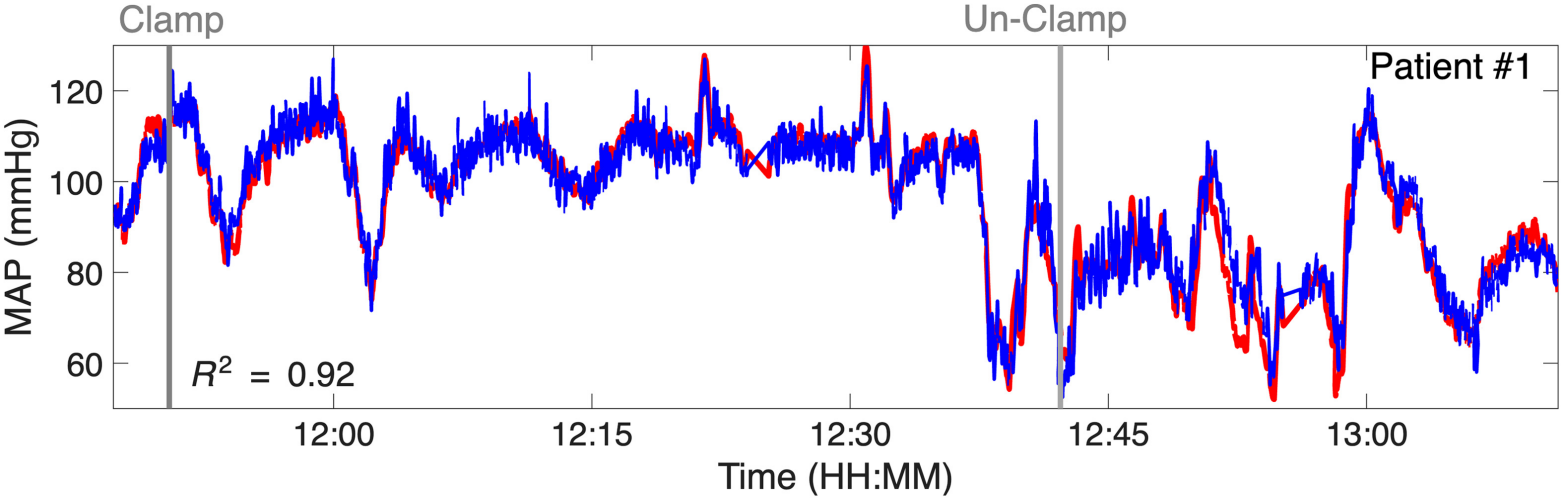
MAP prediction for patient #1 on the contralateral side of surgery using the linear regression model. The red line represents the actual MAP measurements from the arterial line (reference standard), whereas the blue line shows the MAP predictions from the model. The close alignment of these curves highlights the model’s performance.

Figure 5 illustrates MAP prediction for a single subject (patient 1) using the linear regression model. Measurements were taken from the contralateral side at a 2.8-cm source-detector separation. The model achieved an $R^{2}$ value of 0.92, indicating a strong correlation between predicted and actual MAP values.

**Fig. 6:**
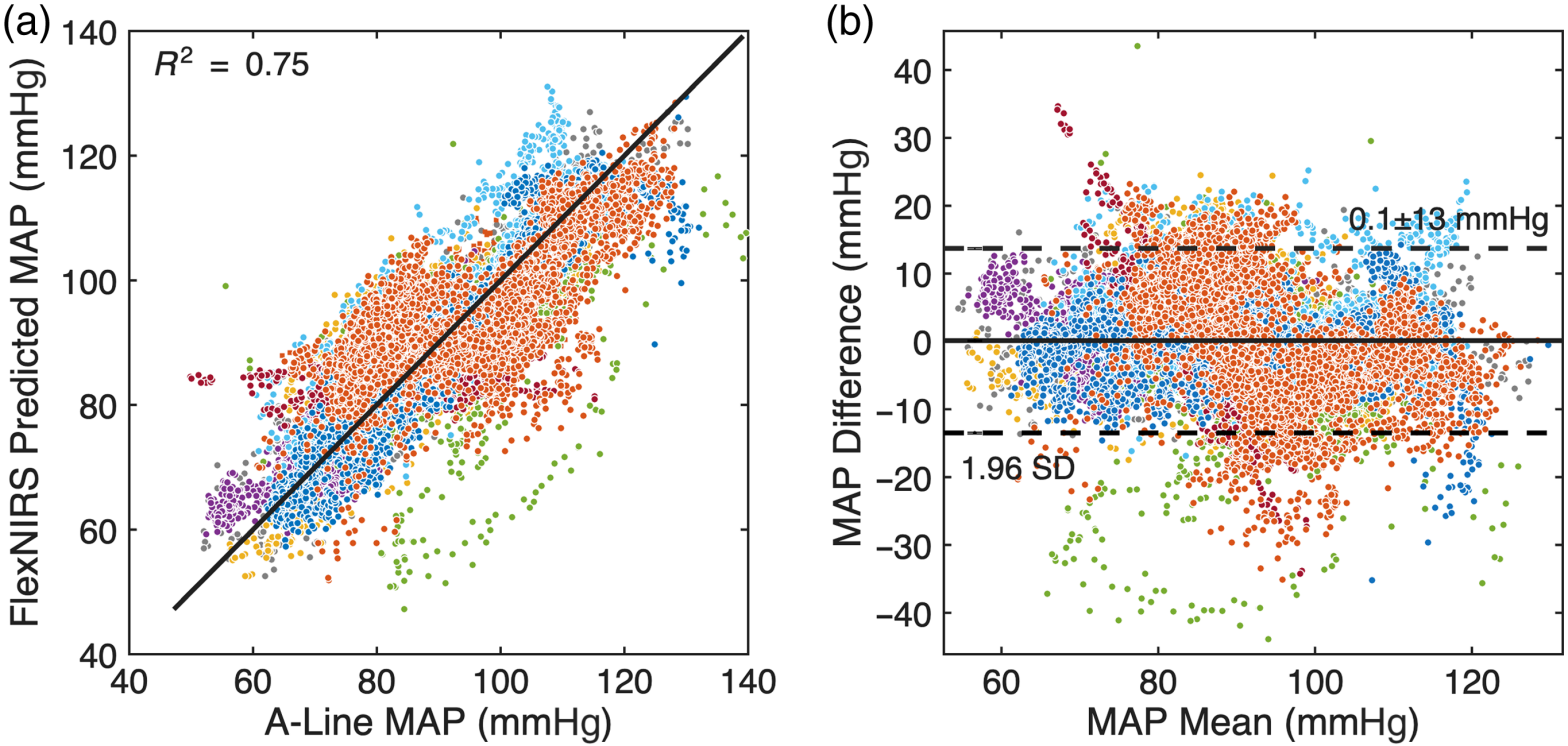
MAP predictions using the linear regression model for nine patients. (a) (Scatter plot): correlation between predicted and actual MAP values using the linear regression model, with an $R^{2}$ value of 0.75. (b) (Bland-Altman analysis): Bland-Altman plot showing a bias of 0.1±13  mmHg for the linear regression model.

Figure 6 presents scatter plots of predicted versus actual MAP values across nine subjects with contralateral-side data, yielding an overall $R^{2}$ value of 0.75. The Bland-Altman analysis for the model demonstrated a bias of 0.1 mmHg, with limits of agreement of ±13  mmHg (1.96 SD).

Table 2 presents the linear regression coefficients for MAP estimation across individual subjects, using P2/P1 and heart rate as predictors. Although both variables were statistically significant (p<0.001) in nearly all cases, the estimated coefficients varied substantially between patients. For example, the P2/P1 coefficient (A) ranged from 19.25 (patient 3) to 143.16 (patient 9), whereas the HR coefficient (B) ranged from 0.24 to 2.52. Notably, in patient #8, P2/P1 was not a significant predictor (p=0.99), indicating inter-individual variability in the physiological relationship between waveform morphology and MAP.

**Table 2:** Patient-specific linear regression coefficients (ε = intercept, A = slope for P2/P1, and B = slope for heart rate) for MAP estimation. Reported values include p values, $R^{2}$, root mean squared error (RMSE), and mean absolute error (MAE) for each subject.

Patient	$\varepsilon_{\left(\text{Intercept}\right)}$ ± SE	$A_{\left(P2/P1\right)}$ ± SE	P ${\text{value}}_{A}$	$B_{\left(\mathrm{HR}\right)}$ ± SE	P ${\text{value}}_{B}$	$R^{2}$	RMSE (mmHg)	MAE (mmHg)
1	−67 ± 1.38	137.10 ± 1.00	<0.001	1.16 ± 0.08	<0.001	0.91	4.60	3.47
3	56.17 ± 4.20	19.25 ± 0.87	<0.001	0.24 ± 0.06	<0.001	0.26	7.34	6.05
4	−16.23 ± 2.33	60.85 ± 1.00	<0.001	0.95 ± 0.03	<0.001	0.56	6.93	5.46
5	−101.28 ± 2.84	87.88 ± 2.19	<0.001	1.66 ± 0.02	<0.001	0.77	5.03	4.36
6	−46.74 ± 2.26	102.62 ± 1.46	<0.001	0.88 ± 0.02	<0.001	0.35	8.47	5.94
7	−80.78 ± 0.93	93.95 ± 0.82	<0.001	2.00 ± 0.01	<0.001	0.80	5.50	3.72
8	54.42 ± 3.98	0.02 ± 5.47	0.99	0.45 ± 0.05	<0.001	0.05	9.46	7.39
9	−179.47 ± 14.07	143.16 ± 0.63	<0.001	2.52 ± 0.17	<0.001	0.89	4.67	3.49
10	−54.26 ± 2.30	66.38 ± 1.07	<0.001	1.48 ± 0.02	<0.001	0.50	9.71	8.06

*SE*, standard error.

### 3.2 MAP Prediction: GPR Model Using Two and Three Features

**Fig. 7:**
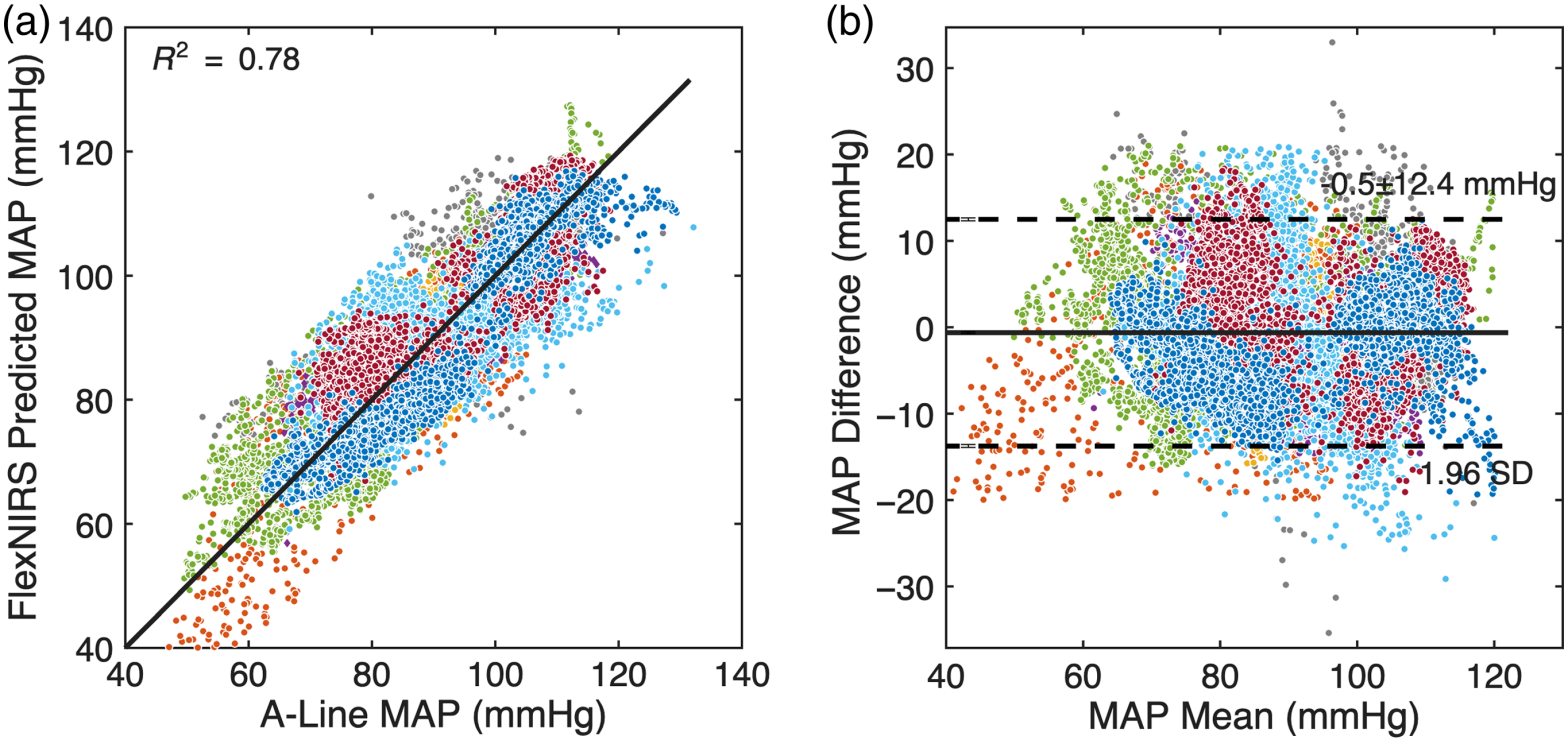
MAP predictions using the two-feature GPR model across nine patients. (a) Scatter plot showing the correlation between predicted and actual MAP values, with an $R^{2}$ value of 0.78. (b) Bland-Altman plot indicating a bias of −0.5 with limits of agreement of ±12.4  mmHg. Different colors represent different patients.

**Fig. 8:**
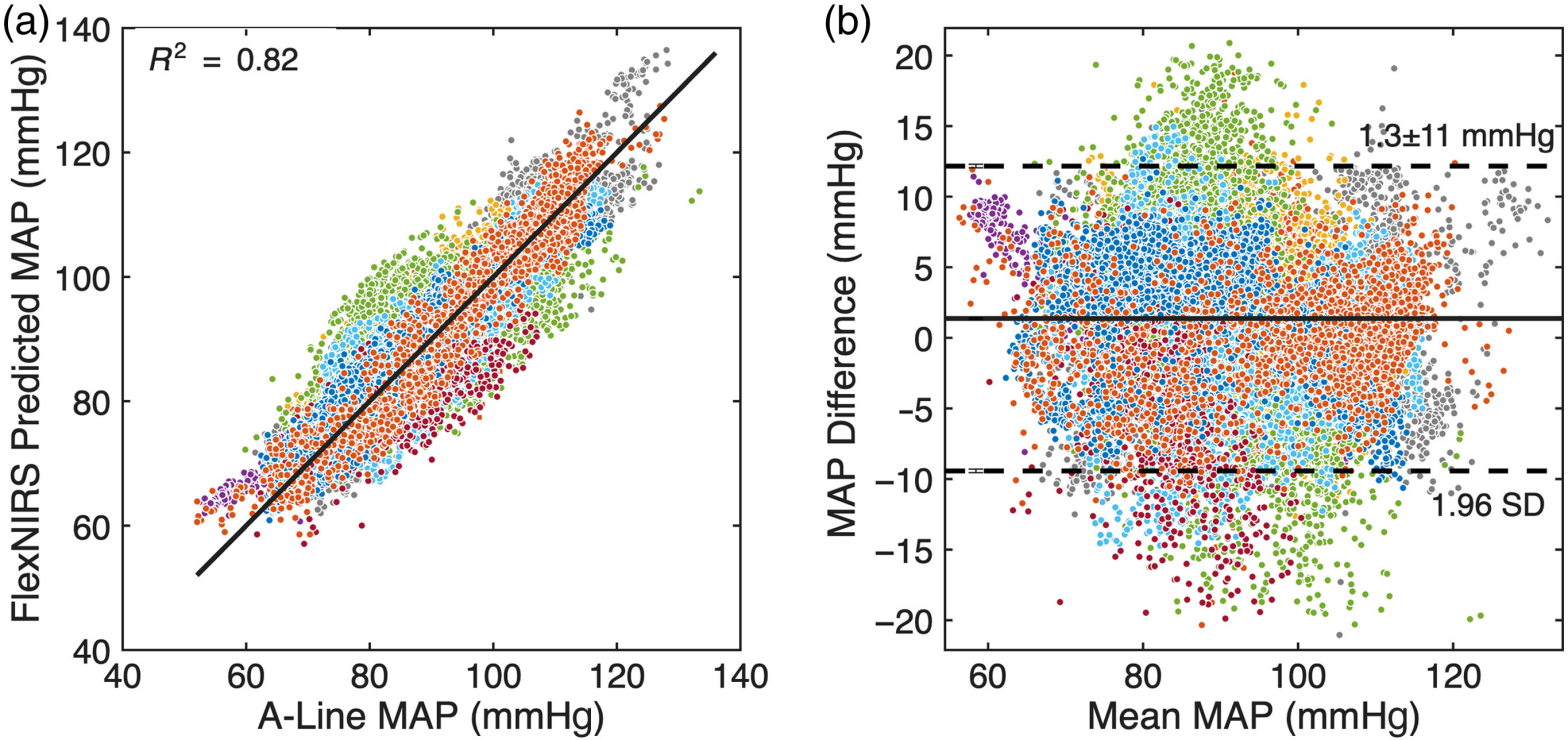
MAP predictions using the three-feature GPR model across nine patients. (a) Scatter plot showing the correlation between predicted and actual MAP values, with an $R^{2}$ value of 0.82. (b) Bland-Altman plot indicating a bias of 1.3 with limits of agreement of ±11  mmHg. Different colors represent different patients.

Figures 7 and 8 show scatter plots comparing predicted and actual MAP values across all subjects using GPR models with two and three features, respectively. Both models were applied to data from the contralateral side at a 2.8-cm source-detector separation. The two-feature model used P2/P1 and HR, whereas the three-feature model included the P2−P1 slope as an additional input.

The two-feature model achieved an $R^{2}$ value of 0.78, indicating a strong correlation between predicted and actual MAP values. The addition of the P2−P1 slope in the three-feature model further improved prediction accuracy, resulting in an $R^{2}$ value of 0.82. This suggests that the inclusion of the slope feature enhances the model’s ability to capture MAP dynamics.

Bland-Altman analysis was used to evaluate agreement between predicted and actual MAP values. The two-feature model exhibited a bias of −0.5  mmHg with limits of agreement of ±12.4  mmHg (1.96 SD). The three-feature model showed slightly higher bias of 1.3 mmHg but narrower limits of agreement (±11  mmHg).

**Fig. 9:**
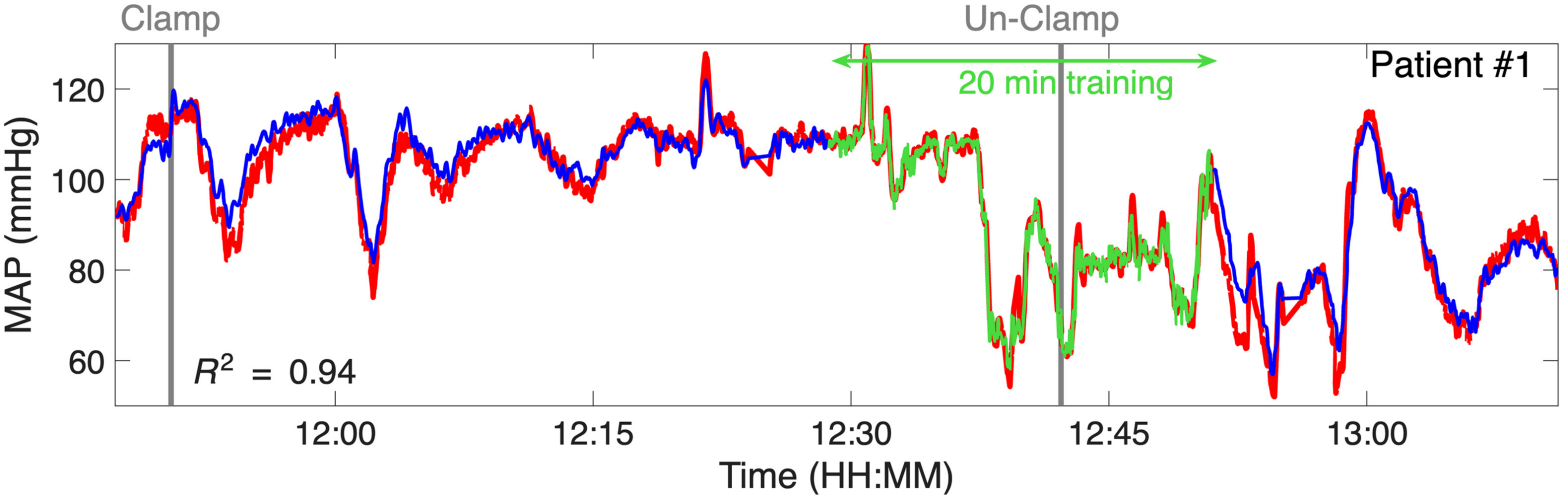
MAP prediction for patient #1 on the contralateral side of surgery using the three-feature GPR model at a source-detector separation of 2.8 cm. The blue line represents continuous MAP predictions from the model, whereas the red line shows actual MAP measurements from the A-line. The green line represents the training period used for GPR model calibration.

Figure 9 provides an example of MAP prediction for a representative subject (patient 1) using the three-features GPR model, visually demonstrating the model’s effectiveness. Time-series MAP estimates for the remaining eight patients with available contralateral data are included in the Supplementary Material.

As summarized in Table 3, the three-feature GPR model outperformed the two-feature model across all evaluation metrics.

**Table 3:** Performance metrics: two-feature versus three-feature GPR models.

Metric	Two-feature model	Three-feature model
R-squared	0.78	0.82
RMSE (mmHg)	7.60	6.53
MAE (mmHg)	4.73	4.05
Limits of agreement (mmHg)	±12.4	±11

The three-feature model outperformed the two-feature model across all metrics. It achieved a higher $R^{2}$ value (0.82 versus 0.78) and lower RMSE and MAE (6.53 and 4.05 mmHg, respectively), indicating improved predictive accuracy. In addition, the narrower limits of agreement (±11.0 versus ±12.4  mmHg) suggest better consistency between predicted and reference values. Normality of the residuals was confirmed, justifying the use of paired t-tests to compare model performance. These tests showed statistically significant improvements with the three-feature model with p=0.0078 for $R^{2}$, p=0.0079 for RMSE, and p=0.0081 for MAE. Results were consistent when using the Wilcoxon signed-rank test, reinforcing the robustness of the findings irrespective of distributional assumptions.

To evaluate how feature sensitivity varies across patients, we analyzed the length-scale parameters learned by the GPR model. These parameters, which govern the sensitivity of the GPR kernel to each input feature, differed substantially across patients (Fig. S2 in the Supplementary Material). The HR feature exhibited the highest degree of variability in length-scale values, including several outliers. One subject with a pacemaker showed the largest HR length scale. By contrast, the P2/P1 feature demonstrated relatively stable length-scale values across subjects. These patterns are further summarized in the supplemental boxplots (Fig. S2 in the Supplementary Material), which illustrate the distribution of length scales for each feature.

### 3.3 Predicting MAP During Surgery: A Comparison of Contralateral and Ipsilateral Sides

The performance of the three-feature GPR model for MAP prediction was evaluated for both contralateral and ipsilateral sides using data from seven patients at a 2.8-cm source-detector separation. Patients 2, 8, and 10 were excluded from the analysis due to missing data on one side (Table 1). By considering this subset of subjects, on the contralateral side, the model achieved an $R^{2}$ value of 0.83, with a bias of 1.3 mmHg and limits of agreement of ±10.2  mmHg (1.96 SD). On the ipsilateral side, the $R^{2}$ value was slightly lower at 0.80, with a bias of −0.5  mmHg and limits of agreement of ±12.7  mmHg (1.96 SD). A paired t-test comparing prediction errors on the contralateral and ipsilateral sides showed no statistically significant difference (p=0.051), indicating that the GPR model performs similarly on both sides.

### 3.4 Predicting MAP: Effect of Source-Detector Separation on Model Performance

To evaluate the impact of source-detector separations on MAP prediction, we compared model performance using data from both 0.8 and 2.8 cm source-detector separations on the contralateral side in nine subjects. As previously reported, the three-feature GPR model achieved an $R^{2}$ value of 0.82 at 2.8 cm, with a bias of 1.3 mmHg and limits of agreement of ±11  mmHg (1.96 SD). At the 0.8-cm separation, the same model yielded an $R^{2}$ value of 0.74, with limits of agreement of ±13  mmHg. Although the 2.8-cm data showed slightly better performance, a paired t-test on per-subject prediction errors did not reveal a statistically significant difference (p=0.11), suggesting comparable predictive accuracy at both separations.

### 3.5 Predicting Systolic and Diastolic Pressure

We also investigated whether the three-feature GPR model could predict systolic and diastolic blood pressure in addition to MAP using data from nine subjects on the contralateral side at the 2.8-cm source-detector separation. As previously reported, the model achieved an $R^{2}$ value of 0.82 for MAP. For SBP, the model yielded an $R^{2}$ value of 0.78, with limits of agreement of ±15.7  mmHg (1.96 SD). DBP predictions showed similarly strong performance, with an $R^{2}$ value of 0.79 and limits of agreement of ±9  mmHg (1.96 *SD*). A repeated measures ANOVA was performed on per-subject prediction errors to evaluate differences in model performance across MAP, SBP, and DBP. The analysis revealed no statistically significant differences (p=0.42), suggesting that the model achieves comparable accuracy in estimating all three pressure components.

## 4 Discussion and Conclusion

We presented the potential of FlexNIRS for continuous noninvasive BP monitoring. In contrast with peripheral PPG, which is often compromised by vasoconstriction and motion artifacts,^7^^,^^24^ FlexNIRS captures pulsatile changes in relative blood volume from the forehead. This site is supplied by arteries linked to the internal carotid system and is therefore more representative of central pressure.^18^ This advantage is further supported by the brain’s low vascular resistance and tightly regulated perfusion, as well as the forehead’s relative insensitivity to vasoconstriction and motion artifacts.^14^^,^^26^ Our results suggest that, once calibrated to the individual, FlexNIRS provides reliable BP estimates, supporting its feasibility for continuous monitoring in diverse clinical settings.

An important consideration is the potential influence of cerebral autoregulation (CA). CA maintains stable cerebral blood flow against slow changes in systemic pressure (<0.2  Hz) over several seconds, but it is ineffective at the cardiac frequency (∼1  Hz), where pulsatile pressure is transmitted directly.^25^^–^^29^ Moreover, our measurements at 2.8-cm source–detector separation are still dominated by extracerebral tissues, which are not subject to cerebral autoregulation. Thus, the beat-to-beat pulsatile component captured by FlexNIRS is unaffected by CA, further supporting its validity for blood pressure estimation.

To evaluate different modeling strategies for blood pressure estimation from FlexNIRS signals, we compared a subject-specific linear regression model, a two-feature GPR model, and a three-feature GPR model. Although recording durations varied across subjects, this did not appear to affect prediction results (see Fig. 9 and Fig. S1 in the Supplementary Material). In subjects with longer recordings, predictions remained stable over time, suggesting that enforcing a uniform testing segment length across subjects would not meaningfully impact the overall evaluation of model accuracy. All models were calibrated individually for each subject and relied on physiologically motivated features derived from the pulsatile NIRS-PPG waveform.

The linear regression model, using the P2/P1 ratio and HR as predictors, achieved an average $R^{2}$ of 0.75. Both predictors were statistically significant in most subjects (p<0.001), and the model structure remained consistent across cases. However, the regression coefficients varied substantially between individuals, underscoring the importance of subject-specific calibration.

These findings suggest that individual differences in vascular compliance, waveform morphology, and sensor placement strongly influence the relationship between features and BP, making population-wide models unreliable.

To better capture nonlinear dependencies, we applied GPR models. The two-feature GPR model, incorporating the P2/P1 ratio and HR, improved performance over the linear regression model, achieving an average $R^{2}$ of 0.78. Adding the P2–P1 slope as a third feature further improved accuracy, yielding an average $R^{2}$ of 0.82, along with lower RMSE and MAE, and narrower Bland–Altman limits of agreement.

Compared with linear regression, both GPR models consistently delivered higher predictive accuracy and tighter agreement with A-line reference measurements (Figs. 6–8), demonstrating the benefit of nonlinear modeling for capturing subject-specific physiological variability.

The inclusion of the P2–P1 slope contributed meaningful predictive information, capturing additional variance in the target variable and enhancing the precision and stability of the model. The observed improvements were statistically significant, underscoring the value of physiologically informative features in individualized BP estimation.

Further analysis of the GPR kernel hyperparameters revealed substantial variability in the learned length-scale parameters and posterior predictive intervals across subjects. This variability highlights the highly individual-specific nature of the relationship between features and blood pressure, reinforcing the need for subject-specific calibration and limiting the applicability of a single model across individuals. In particular, the slope feature showed the greatest dispersion, suggesting that its influence on the output varies notably between patients. One patient with a pacemaker also exhibited an unusually high length-scale for HR, indicating reduced model sensitivity to that feature in this case. These findings underscore the importance of accounting for individual differences when developing predictive models and suggest that subject-specific or hybrid modeling approaches may be more effective than fully pooled models for certain features.

Three-feature GPR models on both the contralateral and ipsilateral sides reliably predicted MAP, with slightly better performance on the contralateral side ($R^{2}=0.83$ versus 0.80). A paired t-test showed no significant difference in prediction errors, suggesting that FlexNIRS remains effective even in the presence of local hemodynamic disturbances. These findings support the robustness of the model and its physiological features under varying vascular conditions.

In conventional CW-NIRS applications, short-separation regression is often used to remove superficial systemic artifacts. However, this approach is not suitable for our application, as both the short (0.8 cm) and long (2.8 cm) source-detector separations capture physiologically relevant pulsatile signals associated with blood pressure. Applying regression between these channels would risk eliminating meaningful signal components rather than isolating superficial noise.

It is also important to note that, although a 2.8-cm separation is often used in cerebral NIRS, our goal is not to measure brain hemodynamics. At this separation, brain sensitivity is low,^30^ and the dominant signal arises from deeper extracranial vessels such as the frontal and temporal arteries. These vessels are better proxies for central blood pressure and are well-targeted by the NIRS-PPG geometry used in this study.^29^

Rather than combining or regressing across separations, we evaluated the performance of each independently. The longer 2.8 cm distance consistently yielded more accurate BP estimates than the shorter 0.8 cm separation, although the difference was not statistically significant (p>0.05). The improved performance is likely due to enhanced sensitivity to deeper vasculature and stronger pulsatile signals.^31^ However, in our controlled setting with minimal head movement, the short separation also yielded reliable MAP estimates. These findings suggest that short separations can be effective under stable conditions, whereas larger separations may offer greater robustness in dynamic or ambulatory environments. Further studies are needed to validate this under conditions involving movement and postural changes.

Finally, our results show that the GPR model performs similarly for MAP, SBP, and DBP, with no significant differences in prediction accuracy. This suggests that the selected features reflect shared physiological trends across all BP components, rather than being specific to one. Although each parameter was modeled independently in this study, future work could explore multi-output approaches that simultaneously estimate MAP, SBP, and DBP from a common feature set.

Despite promising results, this study has several limitations. First, the sample size was relatively small. Data were acquired from 10 patients undergoing CEA, with one excluded due to missing A-line data. All models were calibrated and evaluated on a per-subject basis, limiting the generalizability of the findings. Larger studies are needed to assess FlexNIRS performance across broader populations and support the development of subject-independent models.

Second, although our primary goal is to enable continuous BP monitoring in hospital applications, the current analysis was restricted to the intraoperative setting. Although this environment enabled continuous high-quality data acquisition and provided a clinically indicated reference (A-line), it does not capture the full range of physiological variability encountered postoperatively or in ambulatory contexts. Extending A-line monitoring into the early recovery period would allow evaluation of model robustness over longer timescales and across different physiological states, including wakefulness, sedation, and sleep. Future studies should also test the device in less controlled, real-world conditions involving motion, postural changes, and home-based use.

Although the mean absolute error (MAE∼13  mmHg) and limits of agreement (LOA=±11  mmHg) exceed the AAMI standard of ±5  mmHg, our results are in line with recent cuffless BP monitoring studies under similarly challenging conditions. For instance, Heimark et al.^32^ reported LoA of [−13.9,+11.4]  mmHg using individualized machine learning models in ICU patients. Given that CEA involves deliberate BP modulation and hemodynamic stress, our observed error range appears clinically acceptable for continuous trend monitoring and early detection of significant BP shifts.

A key barrier to translating this method beyond the surgical suite is calibration. In our study, the A-line provided continuous, high-fidelity ground truth for both training and evaluation. In non-surgical settings, such reference data are not readily available. One potential solution is to calibrate the NIRS-PPG system using A-line data collected during a temporary clinical indication (e.g., surgery or ICU stay), enabling continued noninvasive monitoring after A-line removal. Alternatively, brief calibration using oscillometric cuff measurements could offer a fully noninvasive approach. These strategies were not tested in the current study but represent promising paths toward broader adoption.

Finally, although we focused on interpretable models—linear regression and GPR—to establish a baseline, future work should explore more complex machine learning approaches. Deep learning and ensemble methods may improve robustness and generalizability by capturing nonlinear relationships and inter-subject variability. We are currently investigating these techniques in larger datasets to support the development of clinically deployable models.

In conclusion, this study demonstrates the potential of FlexNIRS combined with GPR for accurate, continuous, and noninvasive blood pressure monitoring. By focusing on physiologically meaningful features extracted from the NIRS-PPG waveform, the approach provides reliable estimates of MAP in real time.

## Supplementary Material

10.1117/1.JBO.30.S2.S23913.s01

## Data Availability

Code and data are available upon request.
