# Supplementary material for: Noninvasive continuous blood pressure prediction using FlexNIRS and machine learning during carotid endarterectomy
Source: J Biomed Opt. 2025 Sep 19;30(Suppl 2):S23913. doi: 10.1117/1.JBO.30.S2.S23913 (PMC12447979; doi:10.1117/1.JBO.30.S2.S23913)
Supplement: Supplementary file 1 [file JBO_030_S23913_SD001.pdf]

Supplementary

MAP Predictions over Time for Eight Patients on the Contralateral Side during CEA Surgery

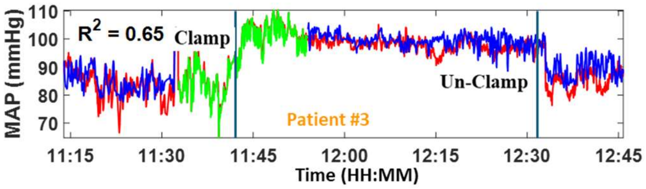

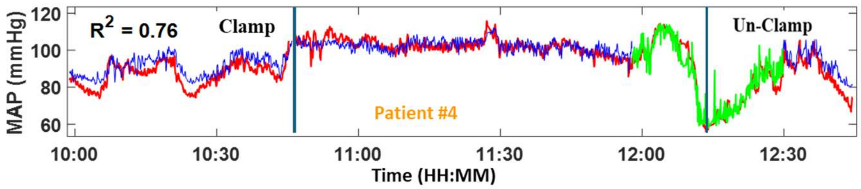

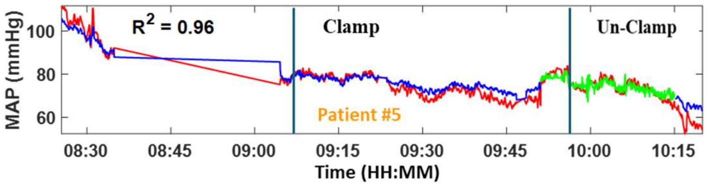

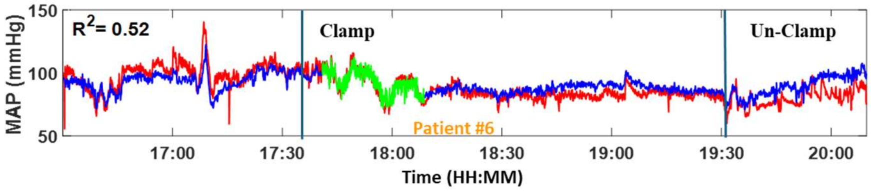

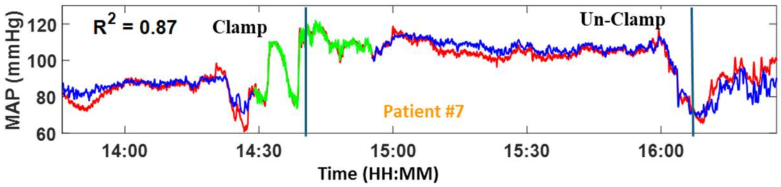

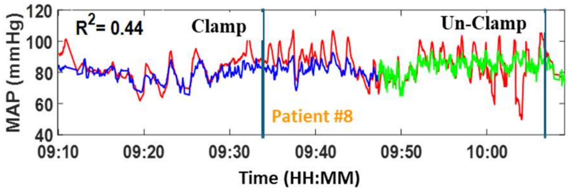

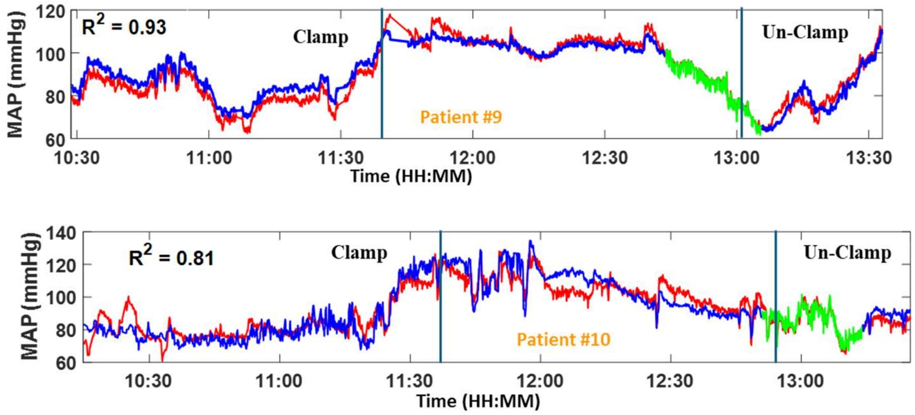

Figure S1: MAP predictions using the GPR model. These plot display the continuous MAP predictions from the GPR model (blue line). The green shaded area represents the training period of the GPR model, and the red line shows the actual MAP measurements from the A-Line.

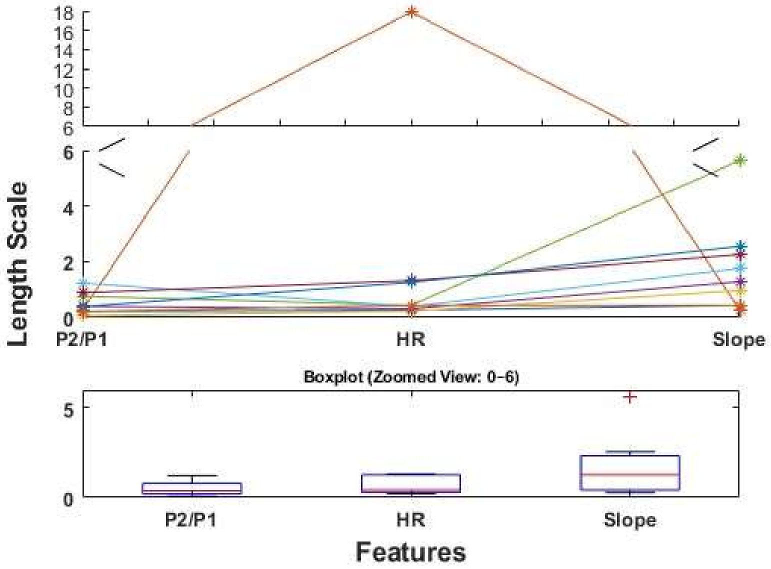

Figure S2. Subject-specific length-scale parameters estimated by GPR for three input features: P2/P1, HR, and Slope. The top two panels display individual length-scale values across patients using a broken y-axis to capture both the main range (0–6) and higher outliers (up to 18), with one subject showing a notably large HR length-scale, corresponding to the presence of a pacemaker. The lower panel presents boxplots summarizing the distribution of length-scales (zoomed to 0–6), showing that Slope exhibits the greatest variability and a prominent outlier, while P2/P1 remains the most stable across subjects. Shorter length-scales indicate higher feature sensitivity within the GPR model.
